# A Comprehensive Review on the Efficacy of Anti-CD20 Therapies in Pemphigus Treatment

**DOI:** 10.7759/cureus.58834

**Published:** 2024-04-23

**Authors:** Arshiya Khan, Adarshlata Singh, Bhushan Madke, Drishti M Bhatt, Shivani D Jangid

**Affiliations:** 1 Dermatology, Jawaharlal Nehru Medical College, Datta Meghe Institute of Higher Education & Research, Wardha, IND; 2 Dermatology, Jawaharlal Nehru Medical College, Datta Meghe Institute of Medical Science, Wardha, IND

**Keywords:** personalized treatment, efficacy, b cell depletion, anti-cd20 therapies, autoimmune blistering disorder, pemphigus

## Abstract

Pemphigus, an autoimmune blistering disorder, poses significant therapeutic challenges due to dysregulated B cells and the involvement of CD20. This review assesses the efficacy of anti-CD20 therapies, including rituximab, ofatumumab, ocrelizumab, and obinutuzumab, in pemphigus treatment. Mechanisms of action, clinical studies, and safety profiles were analyzed, revealing diverse impacts on disease severity. B cell depletion emerged as a pivotal factor, disrupting the autoimmune process and reducing pathogenic antibodies. Varied efficacy and safety profiles among agents underscore the need for personalized treatment strategies guided by biomarkers. Challenges such as resistance and long-term safety concerns necessitate continued research and vigilance. In clinical practice, insights from this review inform nuanced, tailored approaches for improved pemphigus management. The dynamic landscape of emerging therapies and personalized medicine emphasizes the need for ongoing research and strategic clinical decision-making. This review is a foundation for future investigations, providing insights for clinicians and researchers in optimizing pemphigus treatment.

## Introduction and background

Pemphigus is a group of rare autoimmune blistering disorders characterized by the formation of painful, fluid-filled blisters on the skin and mucous membranes. The condition arises from autoantibodies targeting desmogleins, crucial components of intercellular adhesion in the epidermis. Pemphigus can significantly impact patients' quality of life, and its management often poses a therapeutic challenge [1]. B cells play a pivotal role in the immune system, and their dysregulation is implicated in various autoimmune diseases, including pemphigus. In pemphigus, autoreactive B cells produce antibodies that target desmogleins, leading to the loss of cell adhesion and blister formation. CD20, a cell surface antigen present in B cells, has emerged as a therapeutic target for controlling the aberrant immune response in pemphigus [2].

Anti-CD20 therapies are promising treatments designed to modulate B cell activity by selectively depleting CD20-positive B cells. These therapies have gained attention for their potential to alter the course of autoimmune diseases, including pemphigus. Notably, the depletion of B cells is believed to disrupt the autoimmune process and reduce the production of pathogenic antibodies [3]. The purpose of this comprehensive review is to critically examine the efficacy of anti-CD20 therapies in the treatment of pemphigus. By delving into the mechanisms of action, clinical studies, and safety profiles of key anti-CD20 agents, this review aims to analyze their impact on pemphigus management thoroughly. The synthesis of existing knowledge will contribute to a better understanding of the potential benefits and limitations of anti-CD20 therapies, guiding clinicians and researchers in optimizing treatment strategies for individuals with pemphigus.

## Review

Search methodology

The search methodology for this comprehensive review involved a meticulous approach to gathering pertinent literature on the efficacy of anti-CD20 therapies in pemphigus treatment last 10 years. Extensive searches were conducted in reputable academic databases such as PubMed, MEDLINE, Google Scholar, and relevant medical journals until the knowledge. A carefully curated set of keywords and phrases guided the search strategy, including “pemphigus,” “Anti-CD20 therapies,” and the specific names of anti-CD20 agents. The inclusion criteria were well-defined, focusing on studies and clinical trials that explored the mechanisms of action, clinical outcomes, and safety profiles of anti-CD20 therapies in pemphigus. Studies were included if they provided relevant information on the efficacy, mechanisms, or safety of anti-CD20 therapies in pemphigus treatment. Exclusion criteria included studies not written in English, studies not focused on anti-CD20 therapies in pemphigus, and studies lacking sufficient data or methodology details. The synthesis of information aimed to construct a coherent narrative, emphasizing key findings and drawing comparisons among different anti-CD20 therapies. Additionally, the search methodology extended to investigating future perspectives, encompassing emerging therapies and advancements in personalized medicine, by reviewing recent publications and ongoing research. This comprehensive approach provided a robust foundation for understanding anti-CD20 therapies in pemphigus treatment. Characteristics of some of the important studies included in this review are in Table 1.

**Table 1:** Characteristics of some of the important studies included in this review

Authors	Year	Type of Study	Participants	Intervention	Outcome Measures
SCORE (NCT00578305) [4]	2015	Randomized, Placebo-Controlled Trial	Patients with rheumatoid arthritis and inadequate response to methotrexate	Administration of rituximab	Significant efficacy of rituximab in mitigating joint structural damage
Merrill et al. [5]	2010	Clinical Trial	257 participants: Patients entered with >or=1 British Isles Lupus Assessment Group (BILAG) A score or >or=2 BILAG B scores despite background immunosuppressant therapy, which was continued during the trial.	Prednisone was added and subsequently tapered. Patients were randomized at a ratio of 2:1 to receive rituximab (1,000 mg) or placebo on days 1, 15, 168, and 182.	No significant differences were detected between the placebo and rituximab groups regarding primary and secondary efficacy endpoints, including the BILAG-defined response, in the area under the curve and landmark analyses. However, a positive effect of rituximab on the primary endpoint was noted within the African American and Hispanic subpopulations. The safety and tolerability profiles of rituximab were comparable to those of the placebo group.
Aryanian et al. [6]	2021	A retrospective cross-sectional study	999 patients with pemphigus vulgaris received rituximab either as a first-line treatment or after conventional adjuvant therapies.	To compare the side effect profiles of rituximab administered after a course of immunosuppressant agents versus as a first-line therapy	Initiating rituximab treatment early offers advantages to patients with pemphigus, particularly for individuals with mucocutaneous manifestations, pulmonary comorbidities, or a history of smoking, and it decreases their likelihood of experiencing infectious adverse events. Top of Form
Kasperkiewicz et al. [7]	2012	A retrospective, observational study	36 patients with severe pemphigus vulgaris	Administration of rituximab for the treatment of refractory pemphigus	The information gathered from this systematic registry suggests that rituximab is an efficacious and generally safe adjunctive therapy for treatment-resistant pemphigus. To enhance our understanding of the drug's effectiveness and safety profile, there is a need for controlled prospective trials.
Lamberts et al. [8]	2018	Retrospective review	The medical records of 28 patients with pemphigoid diseases	Treatment with rituximab	RTX may be effective in treating stubborn IgG-dominant pemphigoid diseases but shows limited efficacy in conditions predominantly IgA.
Klufas et al. [9]	2020	Case report	A 34-year-old woman with refractory pemphigus vulgaris	Treatment with ofatumumab	Ofatumumab emerges as a promising alternative therapy for pemphigus vulgaris in individuals who exhibit an inadequate response to or intolerance of rituximab.
Izumi et al. [10]	2019	Review of clinical trials	112 studies, including randomized, non-randomized, single-group clinical trials	Treatment with rituximab, ofatumumab ixekizumab, bertilimumab, poly Tregs	Numerous trials exploring novel therapeutic targets indicate that translational research in pemphigus and pemphigoid is rapidly advancing.
Yuan et al. [11]	2022	Review	97 studies were reviewed	Treatment with immunotherapy	Immunotherapy has shown promise in disease control or potential cure for pemphigus, leveraging our current understanding of the immune mechanisms underlying the condition. Top of Form
Marzano et al. [12]	2007	Clinical trial	Six patients with recalcitrant pemphigus were treated	Rituximab was administered intravenously at a dosage of 375 mg/m^2^ body surface once weekly for four weeks.	After six infusions of rituximab, notable improvements were observed in all patients, with the treatment being well-tolerated across the board. However, a significant decrease in anti-desmoglein autoantibodies was only evident in patients with pemphigus foliaceus. Top of Form
Porro et al. [1]	2019	Review	120 studies were reviewed	The treatment was performed with systemic corticosteroids, and immunosuppressive drugs may be associated, including azathioprine and mycophenolate mofetil.	Severe cases of the condition may find relief through corticosteroids administered via intravenous pulse therapy, with recent research indicating the advantageous impact of rituximab, an anti-CD20 immunobiological drug. Pemphigus is a chronic illness associated with a mortality rate of approximately 10%, with septicemia identified as the primary cause of death. Consequently, patients necessitate long-term, multidisciplinary monitoring and care.
Kuriyama et al. [13]	2022	Case report	72-year-old female with paraneoplastic pemphigus	Treatment with obinutuzumab	Paraneoplastic pemphigus (PNP) and bronchiolitis are obliterans (BO). However, exceedingly rare and often fatal complications associated with lymphoid malignancies have shown improvement with a treatment regimen comprising bendamustine and obinutuzumab (BG). This case highlights the potential of BG as a promising therapeutic option for managing PNP and BO, offering hope for better outcomes in these challenging conditions.

Review of anti-CD20 therapies

Rituximab

Rituximab, a chimeric monoclonal antibody, specifically targets the CD20 antigen, predominantly expressed on the surface of pre-B and mature B lymphocytes, enabling its selective lysis of these cells [14]. This targeting mechanism is facilitated through two primary pathways: complement-dependent cytotoxicity (CDC) and antibody-dependent cell-mediated cytotoxicity (ADCC) [15]. In CDC, rituximab binds to specific amino acid regions on CD20, triggering complement system activation and subsequent lysis of CD20+ cells [15]. Similarly, ADCC involves rituximab-induced lysis of CD20+ cells through complement system activation and the engagement of natural killer (NK) cells [15]. Additionally, rituximab exhibits indirect effects by inducing structural changes, apoptosis, and sensitization of cancer cells to chemotherapy, further contributing to its efficacy in treating various B cell malignancies [15]. Notably, rituximab's therapeutic benefits extend to conditions such as non-Hodgkin's lymphoma, chronic lymphocytic leukemia (CLL), and pemphigus vulgaris [14]. Rituximab, an anti-CD20 monoclonal antibody, has gained FDA approval for treating various conditions, encompassing non-Hodgkin lymphoma, CLL, granulomatosis with polyangiitis, and microscopic polyangiitis. Furthermore, the FDA has endorsed rituximab for managing pemphigus vulgaris, suggesting it as a primary treatment for severe cases [16,17]. Rituximab can be administered via intravenous (IV) or subcutaneous (SC) routes. Recent studies indicate comparable clinical effectiveness between SC and IV rituximab, with the SC route offering advantages such as faster drug delivery, reduced potential for dosage errors, and minimized drug wastage [14]. Consequently, rituximab is viable for administration via both IV and SC routes, with the latter providing benefits such as expedited delivery and decreased wastage [14].

Ofatumumab

Ofatumumab, a monoclonal antibody, binds to CD20, a molecule expressed on the surface of healthy and leukemic B lymphocytes. Its mechanism of action encompasses several pathways. Firstly, through ADCC, ofatumumab triggers cell lysis by binding to CD20, rendering B cells susceptible to immediate and delayed lysis by NK cells [18,19]. Additionally, ofatumumab induces cell lysis via CDC, wherein it activates the complement system, destroying CD20-positive B cells [20]. Furthermore, in patients with CLL, ofatumumab facilitates B cell depletion in peripheral blood, contributing to its efficacy in treating this condition [21]. Ofatumumab's multifaceted mechanism of action renders it an effective treatment for various conditions, including CLL and relapsing forms of multiple sclerosis (MS). Administered via subcutaneous injection, ofatumumab has demonstrated superiority over some alternative treatments in reducing the annual relapse rate and decelerating disability progression in MS [21]. Its efficacy across these conditions underscores its therapeutic versatility and potential to improve patient outcomes.

Ocrelizumab

Ocrelizumab, a second-generation anti-CD20 recombinant monoclonal antibody, is utilized in managing and treating primary progressive and relapsing MS [22]. Its therapeutic efficacy is attributed to several mechanisms: Firstly, ocrelizumab, a humanized monoclonal antibody, selectively targets CD20-expressing B cells, which are believed to play a pivotal role in the pathogenesis of MS [23,24]. CD20, a protein present on the surface of specific B cells, is implicated in the disease process [24]. Secondly, ocrelizumab is thought to affect MS by reducing the population of B cells expressing the CD20 protein [24]. This process involves ADCC, wherein the antibody activates other immune cells to attack the myelin insulation and support around nerve cells, consequently releasing inflammatory chemicals in the brain and spinal cord [24]. Additionally, ocrelizumab is believed to induce complement-mediated lysis, which destroys CD20-expressing B cells [25]. Although the exact mechanism of action remains incompletely understood, ocrelizumab is thought to function by selectively targeting and eliminating CD20-expressing B cells, which are implicated in the pathogenesis of MS [23-25].

Obinutuzumab

Obinutuzumab, a humanized type II anti-CD20 monoclonal antibody, features a glycoengineered Fc portion that enhances its affinity for FcγRIIIa receptors on immune effector cells, such as neutrophils and macrophages [26]. Upon binding to CD20 on B cells, obinutuzumab activates immune effector cells, including neutrophils and macrophages [26,27]. Moreover, obinutuzumab directly triggers intracellular death signaling pathways and induces direct cell death [27,28]. Additionally, it can activate the complement cascade, further contributing to the destruction of target cells [27,29]. In contrast to rituximab, a type I anti-CD20 antibody, obinutuzumab operates through a distinct mode of action characterized by increased direct cell death induction and enhanced ADCC and antibody-dependent cellular phagocytosis (ADCP) [28]. This unique mechanism is attributed to its glycoengineering and type II mechanism, which augment ADCC/ADCP while reducing CDC [28]. Overall, obinutuzumab's mechanism of action involves engaging immune effector cells, directly activating intracellular death signaling pathways, and activating the complement cascade, leading to the elimination of target B cells [26-28].

Considerations for Personalized Treatment

Personalized medicine represents a novel medical paradigm that categorizes patients into distinct groups and tailors practices and interventions based on individual predicted responses or risk factors [30,31]. In the context of pemphigus, personalized medicine can be achieved through the identification and cloning of various pemphigus/pemphigoid autoantigens, forming the foundation of current molecular-based diagnostics that enable the differentiation of approximately a dozen pemphigus and pemphigoid variants [30]. Based on lesional histopathology, this differentiation is crucial as patients with pemphigus typically require more aggressive immunosuppressive therapy than those with pemphigoid [30]. Furthermore, a highly personalized treatment approach for pemphigus vulgaris is currently in development, leveraging the selective targeting of autoreactive B cells through the chimeric antigen receptor (CAR) T cell technology [30]. Moreover, rituximab, an anti-CD20 monoclonal antibody, has received FDA approval to treat pemphigus vulgaris in adults [32]. Rituximab has effectively managed refractory pemphigus vulgaris, improving laboratory and clinical outcomes [31,33]. A case-control trial revealed enhanced laboratory and clinical outcomes in patients treated with rituximab compared to those receiving mycophenolate mofetil or azathioprine [34]. However, the response of mucous membranes and cutaneous folds may exhibit delays [33]. Despite this, rituximab is generally safe and well-tolerated, with infusion-related reactions being the most common adverse reaction observed [31,33]. Therefore, personalized medicine in pemphigus can be actualized through the identification and cloning of several pemphigus/pemphigoid autoantigens, alongside the effective utilization of rituximab as an approved treatment option for pemphigus vulgaris in adults.

Challenges and limitations

Resistance to Anti-CD20 Therapies

Resistance to anti-CD20 therapies poses a significant challenge in managing various B cell disorders, including pemphigus. One key contributor to resistance is the reduced expression of CD20 on malignant B cells, a phenomenon influenced by diverse factors and a common cause of resistance to anti-CD20 monoclonal antibodies [35]. Insufficient CD59 or factor H expression on malignant B cells can confer relative resistance to anti-CD20 therapy [3]. Resistance may also arise in cases where ADCC is less effective, often associated with specific FcγRIII polymorphisms [35]. Furthermore, certain anti-CD20 therapies, such as rituximab, have the potential to deplete normal CD20-positive B cells, leading to a loss of CD20 antigens and subsequent resistance to CD20-targeted therapies [36]. The loss of CD20 antigens may be exacerbated by the production of CD20-negative tumor cells, rendering patients resistant to CD20-targeted therapies, including CAR T cell therapy [4]. Despite these challenges, personalized medicine approaches have emerged, involving identifying and cloning various pemphigus/pemphigoid autoantigens. This advancement enables the differentiation of different pemphigus and pemphigoid conditions and facilitates the development of more targeted and effective treatments, such as rituximab, for refractory pemphigus vulgaris [11].

Long-Term Safety Concerns

Recent research has brought attention to long-term safety concerns associated with anti-CD20 therapies. Dr. Zecca and Dr. Gobbi argue that prolonged treatment with anti-CD20 monoclonal antibodies poses an increasingly heightened risk of adverse events [37]. These concerns are further underscored by a study examining the long-term immunological effects of anti-CD20 therapies on humoral responses to COVID-19 vaccines in patients with MS, raising safety concerns regarding the potential impact of anti-CD20 therapies on COVID-19 severity [38]. Although anti-CD20 monoclonal antibodies are currently utilized as maintenance treatments, they are accompanied by long-term safety considerations, including the risk of adverse events and their potential influence on humoral responses to vaccinations [38,39]. Healthcare providers must carefully evaluate the long-term safety profile of anti-CD20 therapies when considering their use in treating B cell disorders, such as pemphigus. These findings underscore the necessity for ongoing research and monitoring of the long-term safety and efficacy of anti-CD20 therapies to ensure that the benefits of these treatments outweigh the potential risks, particularly in the context of extended use and maintenance therapy.

Other Challenges in Pemphigus Treatment

The treatment of pemphigus presents several challenges, encompassing insufficient therapeutic effects, side effects, and the risk of relapse [40,41]. While systemic corticosteroids remain the cornerstone treatment for pemphigus vulgaris, their prolonged use can result in adverse events [40]. Azathioprine and mycophenolate mofetil serve as the first-line steroid-sparing treatments [40]. Rituximab is an effective option for refractory pemphigus vulgaris; however, its long-term safety profile is still being investigated [40,41]. Another hurdle in pemphigus treatment is diagnosis, which can be challenging due to symptom similarities with other skin conditions like lichen planus and lupus erythematosus [42]. Personalized medicine approaches, such as identifying and cloning several pemphigus/pemphigoid autoantigens, have differentiated various pemphigus and pemphigoid conditions [31]. This advancement has facilitated the development of more targeted and effective treatments, such as rituximab, for refractory pemphigus vulgaris [42]. Despite these strides, challenges persist in pemphigus treatment, including insufficient therapeutic effects, side effects, the risk of relapse, and diagnostic difficulties. Personalized medicine strategies and targeted treatments, like rituximab, promise to enhance the efficacy and safety of pemphigus treatment. However, further research is warranted to address these challenges and improve the long-term outcomes of pemphigus patients.

Future perspectives

Emerging Anti-CD20 Therapies

Emerging anti-CD20 therapies hold promise in addressing the limitations of current treatments. Studies indicate that developing novel monoclonal antibodies (mAbs) targeting antigens beyond CD20 and designing mAbs with enhanced effector function could improve treatment outcomes [43]. Introducing mAbs like obinutuzumab, which are less impacted by lower levels of CD20 expression on the cell surface, represents a significant advancement [35]. Moreover, ongoing research and the development of new anti-CD20 mAbs offer numerous advantages over existing treatments, with potential implications for clinical practice [44]. These advancements in anti-CD20 therapies provide hope for overcoming resistance and enhancing treatments' long-term efficacy and safety for B cell disorders, including pemphigus. However, further research and well-designed clinical trials are necessary to fully comprehend the potential of these emerging therapies and their application in personalized treatment approaches. Comparative analyses of the efficacy and safety profiles of the included anti-CD20 drugs- rituximab, ofatumumab, ocrelizumab, and obinutuzumab- are presented in Table 2.

**Table 2:** Comparing the efficacy and safety profiles of the included anti-CD20 drugs: Rituximab, ofatumumab, ocrelizumab, and obinutuzumab

Criteria	Rituximab	Ofatumumab	Ocrelizumab	Obinutuzumab
Mechanism of Action	B cell depletion	B cell depletion	B cell depletion	B cell depletion
Clinical Studies	Extensive research with proven efficacy in pemphigus	Limited studies, but promising results in pemphigus	Limited studies, with mixed results in pemphigus	Emerging data showing potential in pemphigus
Efficacy	Demonstrated efficacy in reducing disease severity	Efficacy supported, though further research is needed	Variable efficacy, depending on the study	Emerging evidence of efficacy in pemphigus
Safety Profile	Generally well-tolerated with common side effects	Generally well-tolerated, with some infusion reactions	Common side effects observed, including infusion reactions	Safety profile being established, with ongoing research
Resistance	Occasional development of resistance	Limited data on resistance	Resistance reported in some cases	Resistance potential under investigation
Long-term Safety Concerns	Some concerns, especially related to prolonged B cell depletion	Limited long-term safety data	The long-term safety profile is yet to be fully elucidated	Ongoing monitoring for long-term safety concerns

Combination Therapies

Several emerging therapeutic approaches hold promise in the management of pemphigus. Bruton's tyrosine kinase inhibitors (BTK inhibitors) have demonstrated potential when used alone or in conjunction with conventional treatments [45]. Additionally, cytokine inhibitors target specific immune system proteins implicated in pemphigus pathogenesis, presenting novel treatment avenues [46]. Anti-CD25 monoclonal antibodies targeting B cells are being explored as potential therapies for pemphigus [46]. Autologous hematopoietic stem cell transplantation has been investigated as a treatment for refractory pemphigus, although further research is necessary to establish its safety and efficacy [46]. Moreover, researchers have engineered chimeric autoantibody receptor (CAAR)-T cells that recognize desmoglein (Dsg) domains, offering a potential precision therapy for pemphigus in the future [46-48]. Personalized medicine approaches aim to enhance treatment efficacy by tailoring therapies to individual patients based on their genetic makeup and disease characteristics [31]. The future of pemphigus treatment will likely involve combination therapies and more personalized approaches, capitalizing on these emerging therapies and strategies to improve disease management and provide new hope for patients. However, additional research is essential to validate the safety and efficacy of these novel treatments.

## Conclusions

In conclusion, the comprehensive review on the efficacy of anti-CD20 therapies in treating pemphigus has shed light on significant findings with implications for both research and clinical practice. The diverse mechanisms of action exhibited by key agents, such as rituximab, ofatumumab, ocrelizumab, and obinutuzumab, underscore the complexity of immune modulation in pemphigus. B cell depletion, a common thread among these therapies, emerges as a critical factor in disrupting the autoimmune process and reducing pathogenic antibody production, leading to positive therapeutic outcomes. The observed variations in efficacy and safety profiles among these agents highlight the need for a personalized treatment approach, emphasizing the importance of identifying biomarkers and patient characteristics to guide treatment selection. The identified challenges, including resistance and potential long-term safety concerns, underscore the necessity for ongoing research and vigilance in clinical practice. As we navigate the dynamic landscape of pemphigus treatment, the insights from this review provide a foundation for future investigations, informing clinicians about the current state of knowledge and guiding strategic decisions for the optimal management of individuals with pemphigus.
